# Hydraulic Strategy of Cactus Root–Stem Junction for Effective Water Transport

**DOI:** 10.3389/fpls.2018.00799

**Published:** 2018-06-12

**Authors:** Hyejeong Kim, Kiwoong Kim, Sang Joon Lee

**Affiliations:** Department of Mechanical Engineering, Pohang University of Science and Technology, Pohang, South Korea

**Keywords:** cactus root, water transport, hydraulic conductivity, root-stem junction, X-ray microimaging

## Abstract

Cactus roots function as a hydraulic safety valve by conducting available water quickly and preventing water loss under drought condition. In particular, the root–stem (R–S) junction is responsible for effective water transport by direct coupling of the water absorptive thin roots and the moisture-filled bulky stem. In this study, the morphological features of the R–S junction were observed by using X-ray micro-imaging technique. Their structural and functional characteristics were also elucidated according to a hydrodynamic viewpoint. With regard to the axial water transport through xylem, the R–S junction prevents water leakage by embolizing large-scale vessels with relatively high hydraulic conductivity. In addition, the axial theoretical hydraulic conductivity of xylem vessels from the roots to the stem drastically increases to facilitate water absorption and prevent water loss. The cortex cell layer of a cactus is thinner than that of other plant species. In the viewpoint of radial conductivity, this property can be the hydraulic strategy of the cactus R–S junction to transport water quickly from the root surface into the xylem. These results suggest that the R–S junction functions as a hydraulic safety valve that can maximize water uptake in axial and radial directions at limited rainfall. This junction can also prevent the stem from leaking water under drought condition.

## Introduction

Deserts receive considerably limited rainfall and plants have evolved for a long time to establish a special hydraulic strategy for effective water transport and conservation for survival in adverse desert environments (North and Nobel, 1997; Nobel, 2003). A representative desert plant, cacti, can manage water effectively under drought conditions and their anatomical features related to water uptake, transport, and storage have been investigated in the last decades (North and Nobel, 1997; Dubrovsky and North, 2002). For instance, their needles can collect water droplets from wet air or fog (Ju et al., 2012). The collected water droplets are transported through trichomes on the epidermis and stored in the stem filled with mucilage (Nobel, 2003; Kim et al., 2017). During intermittent rainy days, cactus roots absorb available water quickly and store it in the stem for a long time as provision against continued drought (Lopez and Nobel, 1991; Huang and Nobel, 1993; North and Nobel, 1997). Roots possess a specialized structure called rhizosheath, which is composed of root hairs, soil particles, and mucilage. Rhizosheath enables the cactus roots to absorb a large amount of water rapidly and prevent the water inside the cortex cell from evaporation (Huang and Nobel, 1992).

Although the water collection and storage methods of cacti are well documented, the hydraulic strategies for transporting absorbed water from the roots to stem remain to be elucidated. In transpiring plants, the transport of water absorbed at the roots depends on the water potential gradient from the roots to the leaves because a large negative potential is generated in xylems by leaf transpiration (Taiz and Zeiger, 2010). However, cacti should minimize water loss through leaf transpiration, especially during daytime. The stem volume for water storage is much larger than the area through which transpiration occurs (Nobel and De la Barrera, 2002). Therefore, cacti possess no sufficient negative water potential in the stem, which is the main power source to uptake water continuously from the roots to the stem. Nevertheless, the inner part of the stem is maintained under wet condition. Since xylem vessels in the roots are the main channel for water transport from the root to the stem, study on the structural characteristics of xylem vessels adapted to water deficit condition would be helpful to understand the hydrodynamic survival strategy of cacti.

The transition zone from the root to stem is the point at which the size of the xylem conduit drastically increases. If a cactus is assumed as a water conduit, its root possesses much narrower conduit compared with the stem which stores a large amount of water. From a hydrodynamic viewpoint, the hydraulic resistance in xylem conduits decreases, as water moves from the root to the stem. Given that xylem vessels are composed of lignified dead cells, they cannot actively respond to the abrupt change in hydraulic resistance. Thus, the xylem vessels at the root–stem (R–S) junction can be assumed to possess a special morphological structure for effective and safe transport of water from root to stem (Zimmerman and Brown, 1971; Holbrook et al., 2002). However, little is known about the hydraulic survival strategy and transport phenomena in the xylem structures of the R–S junction.

Here, the morphological and functional features of the R–S junction of cacti are experimentally and quantitatively elucidated according to a hydrodynamic viewpoint. Water uptake by a cactus roots is determined mainly by the hydraulic conductivity of the roots; this conductivity is composed of the axial conductivity along xylem and the radial conductivity from the root surface into the xylem (Landsberg and Fowkes, 1978; Blizzard, 1980; Passioura, 1988). The structural characteristics of the R–S junction for the regulation of axial and radial hydraulic conductivities were quantitatively analyzed by directly observing the inner morphological structures of the R–S junction under wet and dried states using synchrotron X-ray micro-imaging technique.

## Materials and methods

### Plant material and growth condition

*Opuntia microdasys* (Lehm.) Pfeiff samples were purchased from a local market. They were grown in soil under air-conditioned environment (average temperature: 24 ± 1.5°C, relative humidity: 30 ± 2.5%), and lighting (13 h days, 450 μmol/m^2^/s photosynthetically active radiation, PAR). They were routinely watered at intervals of 3 ~ 4 weeks.

### Imaging techniques

The morphological structures of 4 ~ 6 month old of *O. microdasys* were analyzed using various advanced bio-imaging techniques. For scanning electron microscope (SEM) imaging, the sliced samples of the *O. microdasys* roots and stems were freeze-dried at −84°C for 24 h using a freeze-drying system (LABCONCO, USA). To avoid the charging effect on non-conducting surfaces, the freeze-dried samples were coated with platinum (SC7640 model, Quorum Technology, UK) for 30 s. The detailed morphological images of the freeze-dried roots were captured with a field emission SEM (JEOL JSM-7401F, JEOL).

X-ray imaging experiment was conducted at the beamline 6C Bio Medical Imaging of Pohang Light Source. The 3D morphological structures of hydrated and freeze-dried roots were directly observed using X-ray micro computed tomography (μ-CT). A 10 × objective lens was attached in front of a sCMOS camera (Andor Zyla, Belfast, UK). The spatial resolution was approximately 0.65 μm/pixel for the field of view of 1.7 × 1.4 mm. The distance from the test sample to the camera was fixed at 50 mm. An experimental model was attached to the sample holder, which was rotated from 0 to 180° at intervals of 1°. The 3D tomographic structures were numerically reconstructed and rendered using the Octopus (inCT) and Amira®; (Visualization Sciences Group) software, respectively.

### Quantitative structural analysis on root xylem vessels

The weighted mean diameter (*W*_*m*_) of a vascular bundle was calculated as the product of the mean diameter and the number of xylem vessels in each class, divided by the total number of xylem vessels in the region of interest: $W_{m}=\overset{n}{\underset{i=1}{\sum }}V_{n,i}\times d_{m,i}/T_{n}$, where *V*_*n, i*_ is the number of vessels in class i, *d*_*m, i*_ indicates the mean diameter of vessels in class i, and *T*_*n*_ is the total number of vessels per visual region of interest.

Hydraulic conductance in axial stems is a function of the anatomical characteristics of xylem vessels, especially vessel dimensions (Vercambre et al., 2002). According to Hagen–Poiseuille's law, the root xylem conduits are assumed as cylindrical tubes, and the flow inside the xylems is assumed as a steady laminar flow. The theoretical hydraulic conductivity of the axial flow passing through xylem bundles can be expressed by $k_{h}=\left(\pi \rho /128\eta \right)\overset{n}{\underset{i=1}{\sum }}d_{i}^{4}$, where *d* is the vessel diameter (m), ρ (= 0.997 × 10^6^ g/m^3^ *at* 25°*C*) and η (= 0.89 mPa·s at 25°*C*) are the density and dynamic viscosity of water, respectively (Ewers and Fisher, 1989; Tyree and Ewers, 1991). The theoretical hydraulic conductivity in the axial root was estimated based on the inner morphological structure of the freeze-dried root and stem, which was visualized by X-ray μ-CT. The mean diameter of the xylem vessels was measured from their X-ray cross-sectional images.

### Data analysis

To examine the statistical significance between the average diameters of xylem vessels and those of embolized xylem vessels, *t*-test was applied to the measured data with the *p* < 10^−4^. The Statistical analysis was performed using a Microsoft Excel® macro (Microsoft Corporation, Redmond, WA). To compare the morphological features of different plants, the experimental data on root diameters and cortex thicknesses of different species of fungi (*n* = 96), grass (*n* = 20), tropical tree (*n* = 23), and temperate tree (*n* = 15) were utilized (Gu et al., 2014; Kong et al., 2014; Shiotsu et al., 2015). The root diameters and cortex thicknesses of five O. microdasys samples were measured. The measured data were averaged, and their standard deviation was used to represent the level of error. By applying a linear regression model to the experimental data, the slopes of the fitting lines were estimated with the *p* < 10^−4^.

## Results

### Inner morphological structure of the hydrated R-S junction

**Figure 1:**
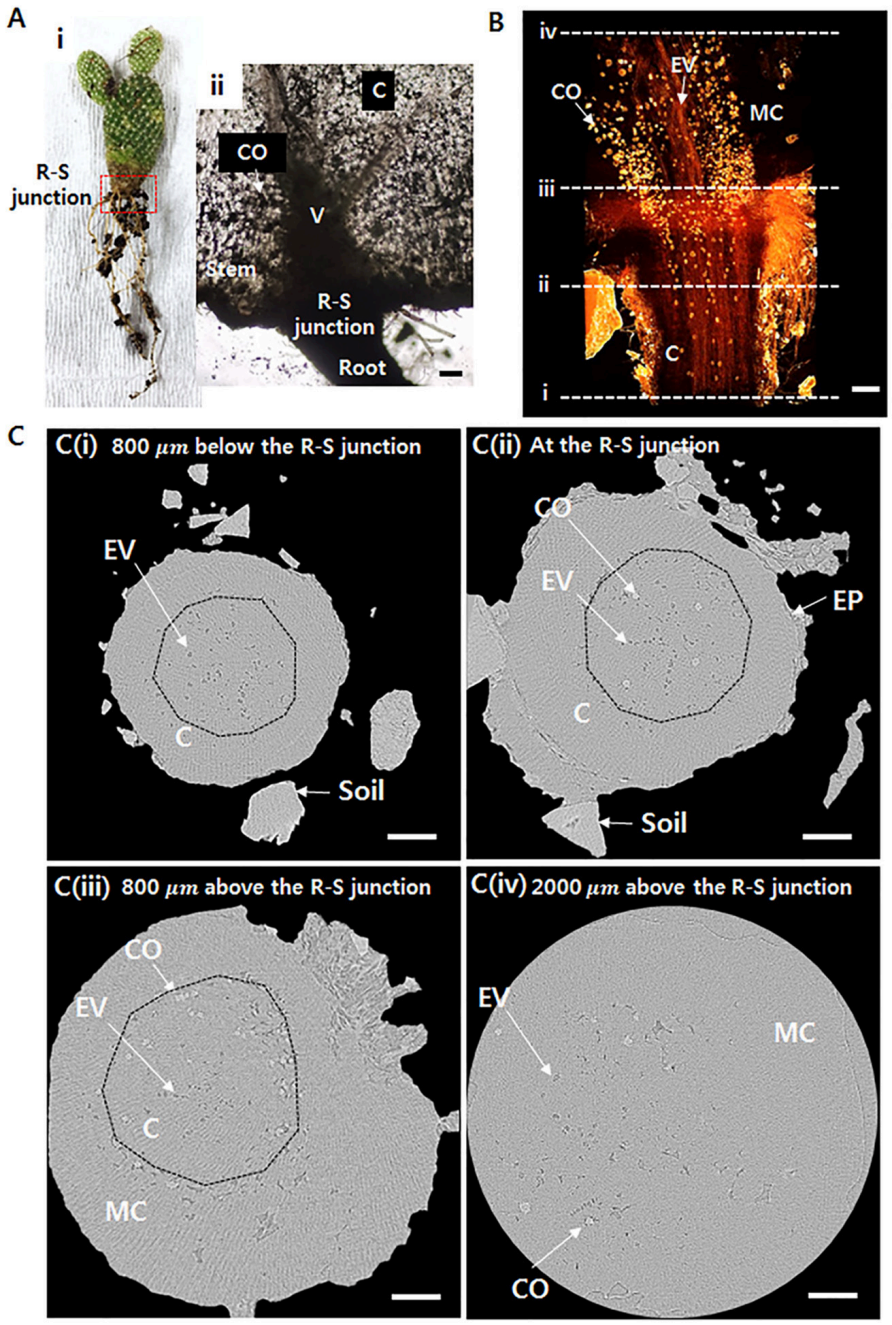
Morphological structures around the root–stem (R–S) junction of *Opuntia microdasys*. **(Ai)** Optical image of *O. microdasys*. Red dotted box indicates the region of the R–S junction. **(Aii)** Optical image of the R–S junction of a sliced *O. microdasys* root. **(B)** 3D image of the *O. microdasys* root visualized by X-ray bioimaging technique. **(C)** Transverse microcomputed tomography (μ-CT) images **(Ci)** at 800 μm below the R–S junction, **(Cii)** at the R–S junction, **(Ciii)** at the stem 800 μm above the R–S junction, and **(Civ)** at the stem 2,000 μm above the R–S junction. These **(Ci–iv)** locations correspond to the dotted lines in **(B)**. R, root; S, stem; CO, calcium oxalate; C, cortex; V, vessel; EV, embolized vessel; EP, epidermis; MC, mucilage cell; dotted lines: endodermis. Scale bar: 200 μm.

Cactus transports the absorbed water from thin roots to a thick stem (Figure 1Ai). When the water transfer path is assumed as a conduit, its radius abruptly increases at the R–S junction (Figure 1Aii). The inner morphological structure of the junction part between the root and stem was observed using X-ray μ-CT (Figures 1B,C). The 3D tomographic image of the R–S junction exhibits spatial distribution of the xylem vessels from the root to the stem (Figure 1B). The embolized vessels filled with air are clearly visible, whereas the water-filled vessels and cells are virtually invisible due to the large attenuation of X-ray beam. The embolized vessels are continuously connected from the root to the stem. Calcium oxalates, appeared as blight spots, are intensively distributed in the stem, especially around xylem vessels. Figure 1C shows the transverse images at locations indicated as dotted lines in Figure 1B. At 800 μm below the R–S junction, not all of the xylem vessels are embolized, and the embolized metaxylem vessels are radially distributed (Figure 1Ci), where dotted lines indicate endodermis). Most protoxylem vessels retain water with high probability. At the R–S junction, some calcium oxalates appear inside the endodermis (Figure 1Cii). With going from the R-S junction to the stem, the cortex cell layer and epidermis are getting thinner and finally do not exist anymore. Then at the position 800 μm above the R-S junction, only the mucilage cells exist in the stem, and lots of calcium oxalates are distributed around the endodermis having numerous air spaces (Figure 1Ciii). At 2,000 μm above the R–S junction, most mucilage cells are filled with water with air spaces (Figure 1Civ).

### Inner morphological structure of the freeze-dried cactus root

**Figure 2:**
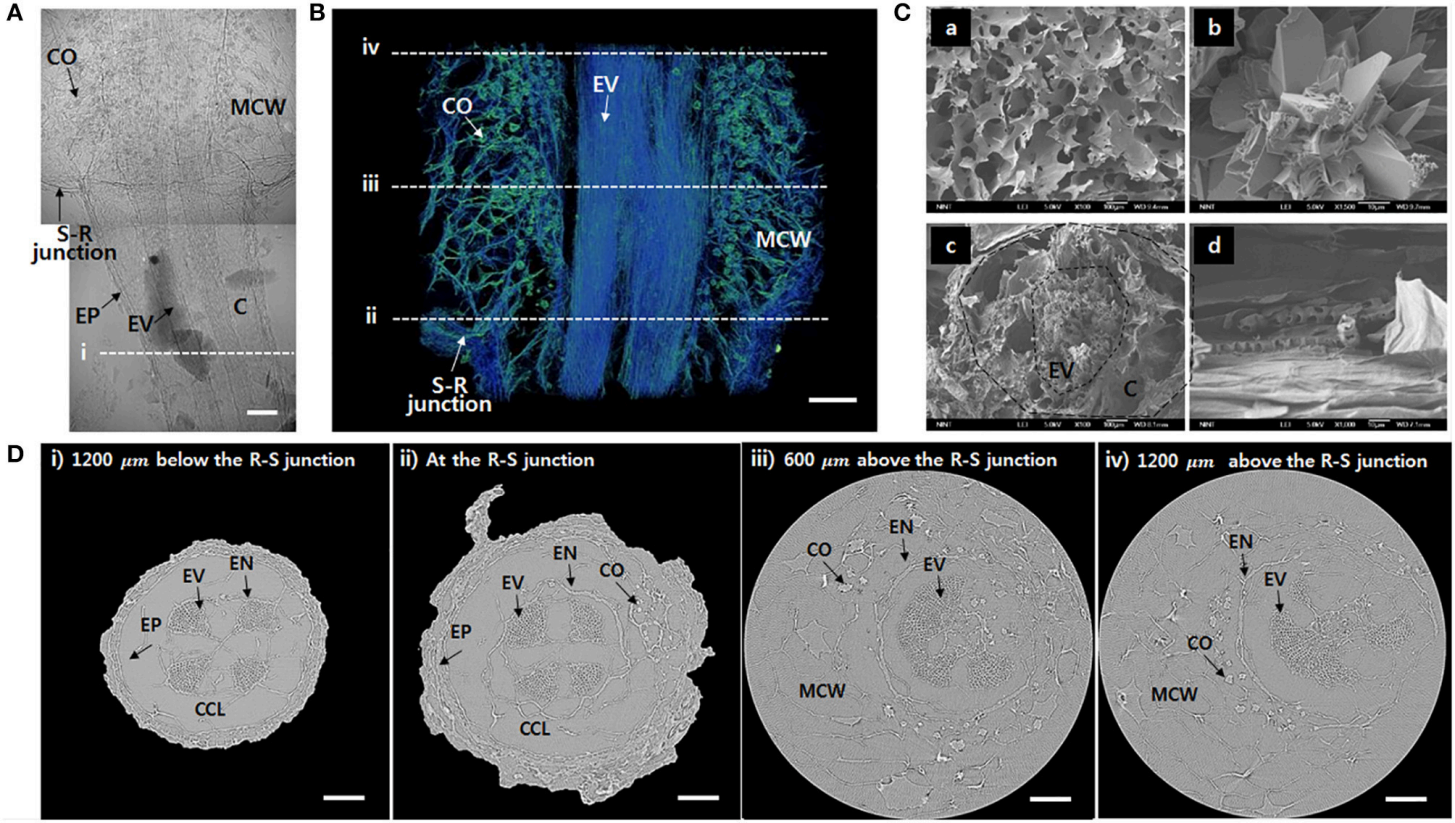
Morphological features of the freeze-dried *O. microdasys* at the R–S junction. **(A)** Typical 2D X-ray image of the freeze-dried *O. microdasys* at the R–S junction. **(B)** 3D image of the R–S junction. **(C)** SEM images of **(Ca)** mucilage cell wall, **(Cb)** calcium oxalate inside the stem, **(Cc)** radial cross-section, and **(Cd)** axial cross-section of the root. **(D)** Transverse μ-CT images **(Di)** at the root 1,200 μm below the R–S junction, **(Dii)** at the R–S junction, **(Diii)** 600 μm above the R–S junction, and **(Div)** 1,200 μm above the R–S junction. These **(Di–iv)** locations correspond to the dotted lines in **(A,B)**. EP, epidermis; MCW, mucilage cell wall; EN, endodermis; CCL, collapsed cortex lacunae. Scale bar: 200 μm.

To observe the xylem vessels in further detail, the test samples were freeze-dried. The xylem vessels with thick lignin cell walls maintain their structure after the freeze-drying procedure. A typical 2D X-ray image captured at the R–S junction shows that the vascular bundles are clearly distinguished inside the roots under the junction (Figure 2A). Nevertheless, inside the stem, the calcium oxalates and mucilage cell walls are complexly overlapped. The 3D reconstructed image provides much clear information about the inner morphological structures (Figure 2B, Movie S1). Straight vessels inside the root are slightly bent in the stem. In addition, mucilage cell walls and calcium oxalates are complexly entangled. Significantly detailed morphologies of the mucilage cells and calcium oxalate inside the stem and the radial cross-section and axial side of the root were observed using a SEM (Figure 2C). Figure 2D shows cross-sectional images at locations indicated as dotted lines (i~ iv) in Figures 2A,B. Xylem vessels are divided into four vascular bundles, and each bundle exhibits a radial fan shape (Figure 2Di). Cortical cells are collapsed during the freeze-drying process, thereby forming cortex lacunae. With approaching from the root to the R–S junction, the diameter of the root increases (Figure 2Dii). Above the R–S junction, the cortex cell layer and epidermis disappear and emerge into the mucilage cells (Figure 2Diii). Inside the stem, mucilage cells distribute as a network, and calcium oxalates surround the endodermis (Figure 2Div). The embolized vessels are radially spread without any regular and definable distribution tendency.

### Morphological features and theoretical hydraulic characteristics

**Figure 3:**
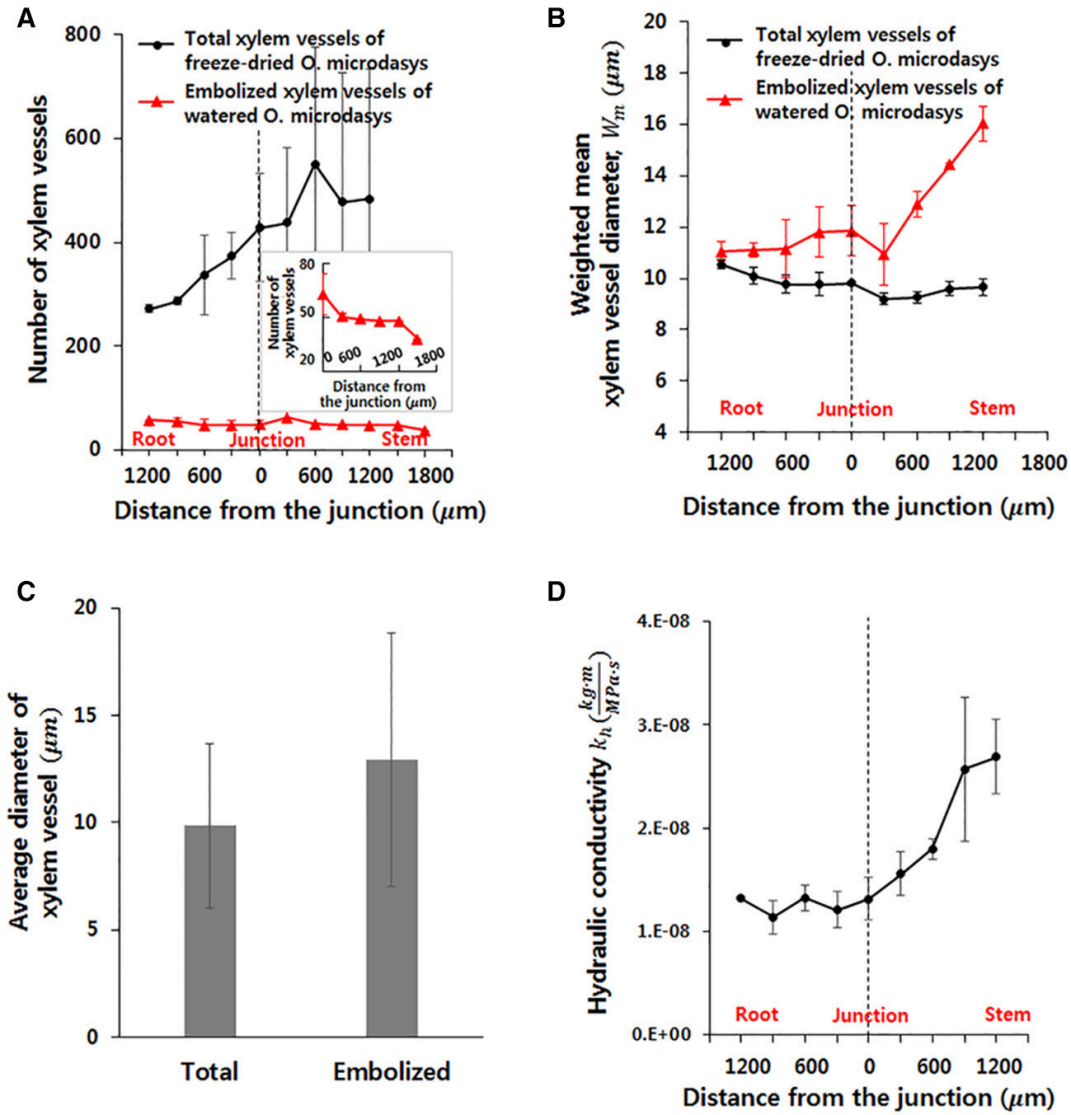
Morphological and hydraulic features of cactus root around the R–S junction. **(A)** Numbers of total and embolized xylem vessels. **(B)** Weighted mean diameters of total and embolized xylem vessels. **(C)** Average diameters of total and embolized xylem vessels. The error bars indicate standard deviations. **(D)** Variation in axial theoretical hydraulic conductivity of the root.

To investigate the hydrodynamic characteristics of the R–S junction, its structural features and theoretical hydraulic conductivity were quantitatively analyzed using the captured X-ray μ-CT images. The total number of xylem vessels in the visual field ranging from 1,200 μm below to 1,200 μm above of the R–S junction largely increases from the root to the stem (Figure 3A). The number increases nearly three times within a short distance of 2,000 μm and subsequently maintains around 500 on average above the R-S junction. Furthermore, the number of embolized xylem vessels is consistently maintained at 50 ± 3 along the axial distance from the root to the stem. The number decreases to approximately 37.5 on average at 1,500 μm above the junction because the embolized vessels are filled with water in the stem, which contains a large amount of water. The weighted mean diameter *W*_*m*_ of total xylem vessels does not show sensitive variation from the root to the stem, as illustrated in Figure 3B. At the position 600 μm below the R-S junction, the *W*_*m*_ of the total xylem vessels (10.12 μm) and that of the embolized vessels (10.58 μm) have similar values. However, with going up from this point to the R-S junction, the *W*_*m*_ of the embolized vessels is larger than that of the total xylem vessels. This value considerably increases in the region beyond the R–S junction. The mean diameter of the embolized xylem vessels is nearly 16 μm at about 1,500 μm above the junction in the stem. Overall, the average diameter of total xylem vessels and embolized xylems is 9.85 and 12.95 μm, respectively (Figure 3C). When we applied *t*-test, the difference between two groups showed statistically significance (*p* < 10^−4^).

Based on the measured diameter and the number of xylem vessels, the theoretical hydraulic conductivity (*k*_*h*_) in the field of view was estimated. The *k*_*h*_ varied along the axial location of xylem vessels (Figure 3D). This values increases slightly without major change in the root region. However, *k*_*h*_ remarkably increases from 1.31 to 2.69 $\left(10^{-8}\cdot \frac{kg\cdot m}{MPa\cdot s}\right)$ after passing the junction with nearly more than two times in the stem region.

### Cortical cell layer for radial water absorption

**Figure 4:**
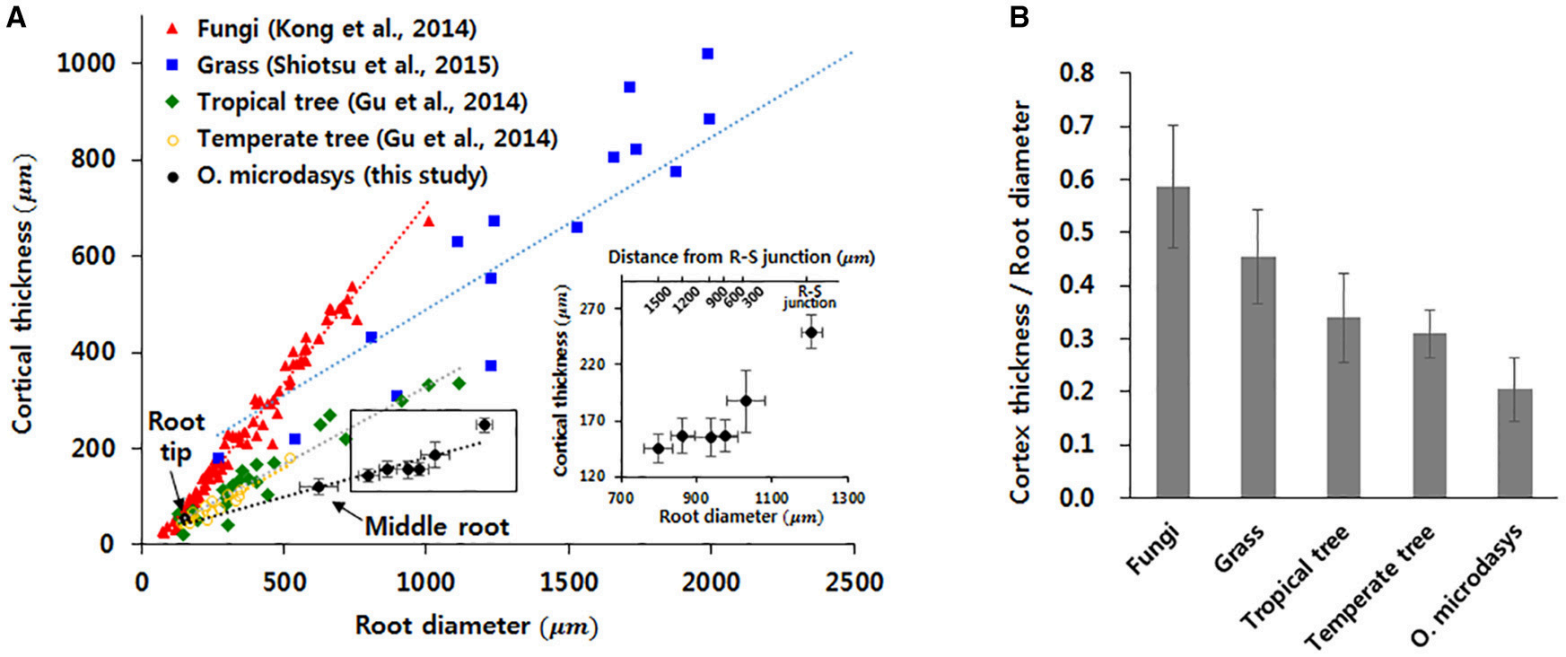
Comparisons of the cortical thickness of cactus root with those of other plant species. **(A)** Variation in cortical thickness according to root diameter. **(B)** Comparisons of the ratios of cortical cell thickness to root diameter with those of other plant species. Data were collected from Gu et al. (2014), Kong et al. (2014), and Shiotsu et al. (2015). The error bars indicate standard deviations.

The thickness of cortical cell layer indicates the distance from the epidermis to the endodermis. For the *O. microdasys* samples, the cortical cell layer is distinctively observed in the root region below the R–S junction. At above the junction, the cortical cell layer becomes invisible when the endodermis gradually merges with mucilage (Figure 2D). Most plant species exhibit positive correlations between the root diameter and the thickness of cortex cell layer (Figure 4A; Gu et al., 2014; Kong et al., 2014; Shiotsu et al., 2015). With assuming that the root diameter and cortical thickness are linearly correlated, the slopes of the fitting lines for several plant species were comparatively estimated by using a linear regression model (*p* < 10^−4^). The slopes of the fitting lines of fungi, grass, tropical tree, and temperate tree are 0.75, 0.36, 0.32, and 0.34, respectively. The slope of cactus root is approximately 0.16, which is about two times smaller than those of other plant species. Therefore, the ratio of the cortical cell thickness to the root diameter of *O. microdasys* is the smallest (Figure 4B). This result indicates that the water absorbed through the *O. microdasys* root hairs takes the shortest pathway to reach xylem vessels compared with those of other plant species.

## Discussion

### Role of xylem structure at the R-S junction on embolism

In the present study, the xylem vessels at the R–S junction of hydrated *O. microdasys* roots were visualized using X-ray μ-CT technique. The captured X-ray CT images provide information on the physical dimension of xylem vessels which play an important role for water transport and maintenance of hydraulic functions of vascular plants (Gullo et al., 1995; Kim and Lee, 2010). The embolized vessels visualized at the R-S junction might be helpful in limiting water loss from the stem under adverse drought condition (Ewers et al., 1992; North and Nobel, 1992). Several xylem vessels with an average diameter of 12.95 μm on average are preferred for embolism. The average diameter of total xylem vessels is approximately 9.85 μm, which is smaller than that of embolized xylem vessels (Figure 3C). In general, the theoretical hydraulic conductivity is proportional to the fourth power of the vessel diameter. Although the average diameter of the embolized xylems is only 1.3 times larger than that of total xylem vessels, the theoretical hydraulic conductivity of the embolized xylems is remarkably nearly three times larger than that of average-sized xylems. The water potential of the cactus roots in dry soil is much smaller than that of the stem filled with watery mucilage, except for short periods immediately after rain. Therefore, reduced hydraulic conductivity due to embolized xylem vessels could reduce water loss by blocking the waterway from the stem to the root. It is different from general plants, where the embolism should be extensively avoided and well repaired for survival with smooth water transport (Hwang et al., 2016).

The embolism formation and its dynamic behavior may be attributed to the conduit size and other functional structures (Brodersen and McElrone, 2013; Lens et al., 2013; Choat et al., 2015; Li et al., 2016). If the relation between the conduit radius and embolism spreading are only considered in a hydrodynamic viewpoint, then the conduit of small radius will be more vulnerable to make the seeded air bubble to induce cavitation inside the conduit. When the seeded bubble increases and reaches the conduit radius, it starts to induce cavitation (Shen et al., 2012). Nonetheless, this hydrodynamic concept does not work in real xylem vessels where large-scale conduits are highly vulnerable to embolization. For instance, the pit membranes in wide conduits may possess large-scale pores, thereby increasing vulnerability to water-stress-induced embolism with high chances of having air-exposed pit membranes (Hargrave et al., 1994).

### Effect of xylem structure at R–S junction on axial flow along xylems

The hydrodynamic conductivity of vascular bundles appears to largely depend on anatomical features, including conduit diameter and number of vessels (Figure 3; Ewers and Fisher, 1989; Tyree and Ewers, 1991; McCulloh and Sperry, 2005). According to the Poiseuille's law, the volume flow rate (*q*) in a xylem vessel can be expressed as follows: ${}_{h}\cdot \left(\frac{d{\text{Ψ }}_{a}}{dl}\right)$, where Ψ_*a*_ is the water potential at a certain location *a*, and *l* represents the length of the xylem vessel. The potential gradient at location *a*, which is the main driving force of water transport, determines the flow direction of water transport. In transpiring plants, a large negative potential is generated at plant leaves by evaporative water, which continuously induces water transport from the soil to leaves (Taiz and Zeiger, 2010). However, cactus minimizes the leaf transpiration to prevent water loss from the stem filled with hydrated mucilage (Nobel and De la Barrera, 2002; Kim et al., 2017). Therefore, even under a harsh drought condition, the water potential in the stem nearly holds the values for wet conditions. Nobel et al reported that the average water potential of cactus stem, Ψ_*stem*_, is −0.67 MPa at 10 h after watering and −0.68 MPa after stopping the water supply for 12 days. When the drought state continues, the water potential of soil becomes highly negative. If the water potential of the stem becomes higher than that of the soil, then water may leak from the stem to the root. Consequently, the cactus should possess a distinct morphological structure as a survival strategy to minimize the water leakage and maximize the water uptake from the roots. In a hydrodynamic point of view, the structural characteristics of the R-S junction would play an important role in managing water transport. With the assumption that the bundle of xylem vessels is a single conduit with an effective radius *W*_*m*_, the hydraulic velocity can be expressed as $v\sim \left\{\frac{{k\;}_{h}}{\pi \cdot {W_{m}}^{2}}\right\}\cdot \left(d{\text{Ψ }}_{a}/dl\right)$, where the water potential gradient is determined from the given environmental condition.

According to the morphological characteristics of the R–S junction, the *k*_*h*_ value remarkably increases from 1.31 to 2.69 $\left(10^{-8}\cdot \frac{kg\cdot m}{MPa\cdot s}\right)$, whereas the weighted mean diameter *W*_*m*_ tends to gradually decrease from 10.55 ± 0.16 to 9.67 ± 0.3 in a short distance from the root to the stem (Figures 3B,D). Although the difference between *W*_*m*_ values at the root and stem is only around 0.88, this difference is about 8 % larger than the mean xylem vessel diameter of the *O. microdasys* samples. In addition, if the sap flow inside the xylem vessels follow the theoretical hydraulic velocity in a conduit, the hydraulic velocity is inversely proportional to the square of *W*_*m*_. Thus, the small difference in *W*_*m*_ has a great influence on the hydraulic velocity. Consequently, the hydraulic velocity is noticeably increased from the root to the stem.

### The effect of xylem structure at R–S junction on radial flow along the cortex cells

The transport of water from the soil toward the root surface and subsequently into the roots is caused by a water potential gradient (Passioura, 1988). Thus, the flow rate across the root tissues, including epidermis, cortical cell layer, and endodermis, depends on the water potential gradient and the radial hydraulic conductivity of the root. This deduction is obtained from the following equation: *q*_*r*_~*k*_*r*_·(Ψ_*r*_−Ψ_*x*_), where *q*_*r*_ is the radial flux of water, *k*_r_ is the radial hydraulic conductivity of the root (*m*/*s*·*MPa*), and Ψ_*r*_ and Ψ_*x*_ are the water potentials on the root surface and in the xylem, respectively. The thickness of cortical cell layers indicates the traveling distance of the absorbed water from the epidermis to the xylem vessels. As the root hydraulic conductivity is inversely related to the thickness of cortical layer, thin cortex cell layers allow water to reach the xylem vessels quickly from the root surface (Rieger and Litvin, 1999). To collect water quickly, cacti develop a shallow, widespread root system to absorb rainwater percolated through the upper soil surface before the rainwater drains away (Rykaczewski et al., 2016). Accordingly, the thin cortex cell layers of cactus root below the R–S junction can help the root to transport water quickly from the root surface to xylem vessels.

## Conclusion

Under limited rainfall, cactus roots are crucial as a flow regulator by conducting available water quickly and preventing the loss of absorbed water (Lopez and Nobel, 1991; Huang and Nobel, 1993; North and Nobel, 1997). In particular, the R–S junction is responsible for hydraulic regulation by connecting the water-deficient roots and the completely hydrated stem. In this study, the morphological structures and functional features of the R–S junction were experimentally and quantitatively investigated in a hydrodynamic point of view. X-ray micro-imaging technique, which is useful for noninvasive visualization of live root samples without pretreatment (Hwang et al., 2016), was used to visualize the inner structures of the R–S junction under wet and dried states. The mean diameter of the embolized vessels is relatively larger than that of total xylem vessels. This condition may help prevent water leakage by embolizing large-size xylem vessels with the aid of relatively large hydraulic conductivity. In addition, the theoretical hydraulic conductivity of xylem vessels from the root to the R–S junction is remarkably increased. If these structural characteristics actually work as a hydraulic strategy for survival, the R–S junction functions as a hydraulic safety valve to enhance water absorption and prevent water loss. Moreover, with regard to radial water transport, the thickness of the cortex cell layers of cactus is thinner than those of other plant species. In general, the axial hydraulic conductivity of a root is much higher than the radial conductivity (Hose et al., 2000). With this distinctive structural feature, cacti seem to reduce the radial transport and enhance the axial transport. This feature can be another hydraulic strategy to transport water quickly from the root surface to xylem vessels. The adoption of such hydraulic strategies is crucial for the survival of cacti, which store considerable amount of water in their stem under several months of continued adverse drought condition (North and Nobel, 1997; Nobel, 2003). Previous studies on the anatomical inner structures of the cactus R–S junction are scarce. Hence, this study will contribute to the understanding of the plant water relations of cacti. The present results can also provide important information for designing various practical applications, in which one-way water transport under harsh environment is inevitable.

## Author contributions

HK and SL: proposed the study; HK: performed the experiment; HK and KK: processed images and analyzed the experimental data. All authors discussed the results. All authors participated in completing the manuscript.

### Conflict of interest statement

The authors declare that the research was conducted in the absence of any commercial or financial relationships that could be construed as a potential conflict of interest.

## References

[B1] Blizzard W. E. (1980). Comparative resistance of the soil and the plant to water transport. Plant Physiol. 66, 809–814. 10.1104/pp.66.5.809 16661531 PMC440731

[B2] Brodersen C. R. McElrone A. J. (2013). Maintenance of xylem network transport capacity: a review of embolism repair in vascular plants. Front. Plant Sci. 4:108. 10.3389/fpls.2013.00108 23630539 PMC3633935

[B3] Choat B. Brodersen C. R. McElrone A. J. (2015). Synchrotron X-ray microtomography of xylem embolism in Sequoia sempervirens saplings during cycles of drought and recovery. New Phytol. 205, 1095–1105. 10.1111/nph.13110 25385085

[B4] Dubrovsky J. G. North G. B. (2002). Root structure and function, in Cacti: Biology and Uses, ed Nobel P. S. (Berkeley, CA: University of California Press), 41–56.

[B5] Ewers F. W. Fisher J. B. (1989). Techniques for measuring vessel lengths and diameters in stems of woody plants. Am. J. Bot. 76, 645–656. 10.1002/j.1537-2197.1989.tb11360.x

[B6] Ewers F. W. North G. B. Nobelf P. S. (1992) Root—stem junctions of a desert monocotyledon a dicotyledon: hydraulic consequences under wet conditions during drought. New Phytol. 121 377–385. 10.1111/j.1469-8137.1992.tb02937.x 33874157

[B7] Gu J. Xu Y. Dong X. Wang H. Wang Z. (2014). Root diameter variations explained by anatomy and phylogeny of 50 tropical and temperate tree species. Tree Physiol. 34, 415–425. 10.1093/treephys/tpu019 24695727

[B8] Gullo M. A. Salleo S. Piaceri E. C. Rosso R. (1995). Relations between vulnerability to xylem embolism and xylem conduit dimensions in young trees of Quercus corris. Plant Cell Environ. 18, 661–669.

[B9] Hargrave K. Kolb K. Ewers F. Davis S. (1994). Conduit diameter and drought-induced embolism in Salvia mellifera Greene (Labiatae). New Phytol. 126, 695–705. 10.1111/j.1469-8137.1994.tb02964.x

[B10] Holbrook N. M. Zwieniecki M. A. Melcher P. J. (2002). The dynamics of “dead wood”: maintenance of water transport through plant stems1. Integr. Comp. Biol. 42, 492–496. 10.1093/icb/42.3.492 21708743

[B11] Hose E. Steudle E. Hartung W. (2000). Abscisic acid and hydraulic conductivity of maize roots: a study using cell-and root-pressure probes. Planta 211, 874–882. 10.1007/s004250000412 11144273

[B12] Huang B. Nobel P. S. (1992). Hydraulic conductivity and anatomy for lateral roots of agave deserti during root growth and drought-induced abscission. J. Exp. Bot. 43, 1441–1449. 10.1093/jxb/43.11.1441

[B13] Huang B. Nobel P. S. (1993). Hydraulic conductivity and anatomy along lateral roots of cacti: changes with soil water status. New Phytologist 123, 499–507. 10.1111/j.1469-8137.1993.tb03762.x 33874108

[B14] Hwang B. G. Ryu J. Lee S. J. (2016). Vulnerability of protoxylem and metaxylem vessels to embolisms and radial refilling in a vascular bundle of maize leaves. Front. Plant Sci. 7:941. 10.3389/fpls.2016.00941 27446168 PMC4921478

[B15] Ju J. Bai H. Zheng Y. Zhao T. Fang R. Jiang L. (2012). A multi-structural and multi-functional integrated fog collection system in cactus. Nat. Commun. 3:1247. 10.1038/ncomms2253 23212376 PMC3535335

[B16] Kim H. K. Lee S. J. (2010). Synchrotron X-ray imaging for nondestructive monitoring of sap flow dynamics through xylem vessel elements in rice leaves. New Phytol. 188, 1085–1098. 10.1111/j.1469-8137.2010.03424.x 20735745

[B17] Kim K. Kim H. Ho Park S. Joon Lee S. (2017). Hydraulic strategy of cactus trichome for absorption and storage of water under arid environment. Front. Plant Sci. 8:1777. 10.3389/fpls.2017.01777 29093723 PMC5651663

[B18] Kong D. Ma C. Zhang Q. Li L. Chen X. Zeng H.. (2014). Leading dimensions in absorptive root trait variation across 96 subtropical forest species. New Phytol. 203, 863–872. 10.1111/nph.12842 24824672

[B19] Landsberg J. Fowkes N. (1978). Water movement through plant roots. Ann. Bot. 42, 493–508. 10.1093/oxfordjournals.aob.a085488

[B20] Lens F. Tixier A. Cochard H. Sperry J. S. Jansen S. Herbette S. (2013). Embolism resistance as a key mechanism to understand adaptive plant strategies. Curr. Opin. Plant Biol. 16, 287–292. 10.1016/j.pbi.2013.02.005 23453076

[B21] Li S. Lens F. Espino S. Karimi Z. Klepsch M. Schenk H. J. (2016). Intervessel pit membrane thickness as a key determinant of embolism resistance in angiosperm xylem. Iawa J. 37, 152–171. 10.1163/22941932-20160128

[B22] Lopez F. B. Nobel P. S. (1991). Root hydraulic conductivity of two cactus species in relation to root age, temperature, and soil water status. J. Exp. Bot. 42, 143–149. 10.1093/jxb/42.2.143

[B23] McCulloh K. A. Sperry J. S. (2005). Patterns in hydraulic architecture and their implications for transport efficiency. Tree Physiol. 25, 257–267. 10.1093/treephys/25.3.257 15631974

[B24] Nobel P. S. (2003). Environmental Biology of Agaves and Cacti. Cambridge: Cambridge University Press.

[B25] Nobel P. S. De la Barrera E. (2002). Stem water relations and net CO_2_ uptake for a hemiepiphytic cactus during short-term drought. Environ. Exp. Bot. 48, 129–137. 10.1016/S0098-8472(02)00016-3

[B26] North G. B. Nobel P. S. (1992). Drought-induced changes in hydraulic conductivity and structure in roots of Ferocactus acanthodes and Opuntia ficus-indica. New Phytol. 120, 9–19.

[B27] North G. B. Nobel P. S. (1997). Drought-induced changes in soil contact and hydraulic conductivity for roots of Opuntia ficus-indica with and without rhizosheaths. Plant Soil 191, 249–258. 10.1023/A:1004213728734

[B28] Passioura J. (1988). Water transport in and to roots. Annu. Rev. Plant Physiol. Plant Mol. Biol. 39, 245–265. 10.1146/annurev.pp.39.060188.001333

[B29] Rieger M. Litvin P. (1999). Root system hydraulic conductivity in species with contrasting root anatomy. J. Exp. Bot. 50, 201–209. 10.1093/jxb/50.331.201

[B30] Rykaczewski K. Jordan J. S. Linder R. Woods E. T. Sun X. Kemme N.. (2016). Microscale mechanism of age dependent wetting properties of prickly pear cacti (Opuntia). Langmuir 32, 9335–9341. 10.1021/acs.langmuir.6b02173 27537082

[B31] Shen F. Wang Y. Cheng Y. Zhang L. (2012). Three types of cavitation caused by air seeding. Tree Physiol. 32, 1413–1419. 10.1093/treephys/tps089 23100258

[B32] Shiotsu F. Abe J. Doi T. Gau M. Morita S. (2015). Root morphology and anatomy of field-grown Erianthus arundinaceus. Am. J. Plant Sci. 6:103 10.4236/ajps.2015.61012

[B33] Taiz L. Zeiger E. (2010). Plant Physiology, 5th Edn. Sunderland, MA: Sinauer Associates.

[B34] Tyree M. T. Ewers F. W. (1991). The hydraulic architecture of trees and other woody plants. New Phytol. 119, 345–360. 10.1111/j.1469-8137.1991.tb00035.x

[B35] Vercambre G. Doussan C. Pages L. Habib R. Pierret A. (2002). Influence of xylem development on axial hydraulic conductance within Prunus root systems. Trees Struct. Funct. 16, 479–487. 10.1007/s00468-002-0190-6

[B36] Zimmerman M. H. Brown C. L. (1971). Trees: Structure and Function. New York, NY: Springer-Verlag.

